# Spatial Structure, Transmission Modes and the Evolution of Viral Exploitation Strategies

**DOI:** 10.1371/journal.ppat.1004810

**Published:** 2015-04-21

**Authors:** Thomas W. Berngruber, Sébastien Lion, Sylvain Gandon

**Affiliations:** 1 Phage Technology Center GmbH, Bönen, Germany; 2 Centre d'Ecologie Fonctionnelle et Evolutive (CEFE), UMR 5175, CNRS-Université de Montpellier—Université Paul-Valéry Montpellier—EPHE. Montpellier, France; University of Edinburgh, UNITED KINGDOM

## Abstract

Spatial structure and local migration are predicted to promote the evolution of less aggressive host exploitation strategies in horizontally transmitted pathogens. Here we explore the effect of spatial structure on the evolution of pathogens that can use both horizontal and vertical routes of transmission. First, we analyse theoretically how vertical transmission can alter evolutionary trajectories and confirm that space can impede the spread of virulent pathogens. Second, we test this prediction using the latent phage λ which transmits horizontally and vertically in *Escherichia coli* populations. We show that the latent phage λ wins competition against the virulent mutant λcI857 in spatially structured epidemics, but loses when spatial structure is eroded. The vertical transmission of phage λ immunizes its local host pool against superinfection and prevents the spread of the virulent λcI857. This effect breaks down when mixing facilitates horizontal transmission to uninfected hosts. We thus confirm the importance of spatial structure for the evolutionary maintenance of prudent infection strategies in latent viruses.

## Author Summary

Why do some viruses use latent infection strategies and in which environments can such prudent host exploitation strategies evolve? Theory of mathematical epidemiology and social evolution predicts that prudent pathogens can evolve when they cluster in space and share the mutual benefits of prudent exploitation of the host population. Here we extend the theory to study the effect of spatial structure on the evolution of pathogens that can transmit both horizontally and vertically. We explore these effects experimentally by competing the latent bacteriophage λ and the virulent mutant λcI857 in spatially structured epidemics and gradually erode spatial structure. We show that the latent bacteriophage λ only wins in a spatially structured epidemic. Yet, a breakdown of the epidemic structure by long-range transmission reduces the benefit of latency by 500 fold. This demonstrates that long-rage transmission, as for example by air travel, might select for much more virulent types of previously latent viruses.

## Introduction

When individuals compete for a common resource natural selection often favours more aggressive exploitation strategies. This may lead to resource exhaustion and, consequently, to population extinction, a process known as the 'tragedy of the commons'[1]. Because pathogens compete for a common resource (the host population), the same process may select for extreme exploitation strategies. Yet, two main ecological factors can alter this evolutionary outcome and promote the evolution of less aggressive exploitation strategies.

First, epidemic spread reduces the density of susceptible hosts and can feed back on the selective pressure that favours intermediate exploitation strategies [2–5]. Indeed, decreased availability of susceptible hosts weakens selection for higher transmission rates [6–8]. Ultimately, this may select for intermediate evolutionarily stable strategies, balancing the benefit (transmission) and the cost (virulence: induced host mortality) of host exploitation. We recently tested this idea using the bacteriophage λ in experimental epidemics spreading through well-mixed environments [9]. We confirmed that more virulent strains are indeed selected for during early epidemics, when uninfected hosts are abundant, but also that natural selection favours latent strains of the virus as disease prevalence increases.

**Fig 1:**
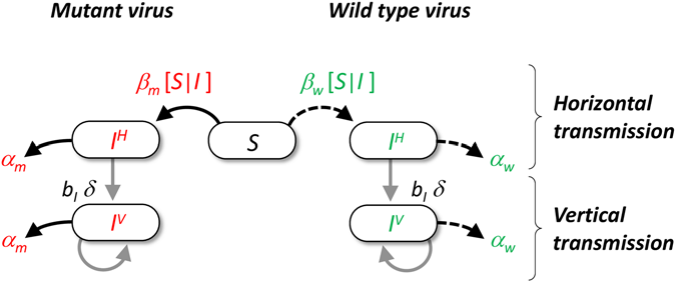
Schematic representation of the pathogen life cycle. In our model we assume that hosts can either be uninfected or infected by the wild type (in green) or a mutant genotype (in red). Transmission can take two different routes. Horizontal transmission occurs between a susceptible host and an infected host with genotype *i* with rate *β*_*i*_[*I*|*S*] (see supporting information Theory in S1 Text). Vertical transmission occurs when and infected host reproduces in an empty site with rate *b*_*I*_ *δq*_*0/i*_, where *b*_*I*_ is the reproduction rate of infected hosts, *δ* is the fidelity of vertical transmission and *q*_*0/i*_ measures the average local density of empty sites around hosts infected with genotype *i*. Among hosts infected by the same genotype we distinguish hosts that got infected horizontally (noted $I_{i}^{H}$) or vertically (noted $I_{i}^{V}$). Both types of hosts can reproduce and have an increased mortality rate *α*_*i*_ (i.e. virulence) due to the infection with genotype *i*.

Second, theoretical studies on spatially structured populations indicate that localized transmission often favours more prudent host exploitation strategies [10–21]. Indeed, when epidemics are spatially structured, extreme strategies of host exploitation may lead to the over-exploitation of the local host supply and therefore fail to invade. The intensity of local competition for susceptible hosts depends, however, on the precise genetic and epidemiological structure of the pathogen population [21]. The effect of spatial structure on the evolution of pathogens has had some experimental support [22–23] which is, however, based on experiments with obligate killing and strictly horizontally transmitting pathogens. Previous experimental work has shown that vertical transmission selects for lower virulence [24–25] but the effect of spatial structure on vertically transmitted pathogens remains an open question. In the present study we explore the effect of spatial structure on the evolution of pathogens that can be transmitted both vertically and horizontally. We first develop and analyse a theoretical model to understand the evolutionary dynamics of pathogens in spatially structured epidemics (Fig 1). This model allows infected hosts to reproduce and to transmit their pathogens vertically. We use this model to study the competition between different pathogen genotypes under various levels of mixing (see supporting information in S1 Text). Then, we confront these predictions with experiments performed with the horizontally and vertically transmitting temperate bacteriophage λ. Bacteriophage λ is an avirulent pathogen that controls its own replication by the virulence repressor *cI* and integrates into the host genome as a prophage without killing the host cell—a process known as lysogenization. The virulence repressor *cI* furthermore controls prophage λ reactivation and excludes superinfection by a second viral particle [26]. Yet, spontaneous mutations in *cI* can markedly increase virulence [27]. We studied competition between temperate bacteriophage λ and its virulent mutant λcI857. Mutant λ*cI857* carries an unstable virulence repressor protein (cI857) that biases the viral life cycle towards horizontal transmission: whereas λ lysogenizes about 60% of newly infected cells killing only about 40% of cell, λcI857 kills 96% of host cells upon first infection (see Fig S2B in S1 Text). In addition λcI857 shows a higher rate of prophage reactivation, sacrificing infected host cells for increased production of free viral particles (Fig S2A in S1 Text). Furthermore, λcI857 is less efficient in the exclusion of superinfection (see Fig S2B in S1 Text). Whereas the temperate λ wildtype shows an avirulent host exploitation strategy the virulent mutant λcI857 readily exploits and kills its host cell. To experimentally investigate the effect of spatial structure on the success of these opposing strategies we competed λ and λcI857 in experimental epidemics with different degrees of spatial structure (Manipulation of spatial structure is described in Material & Methods and [28]).

## Results

### Theory

**Fig 2:**
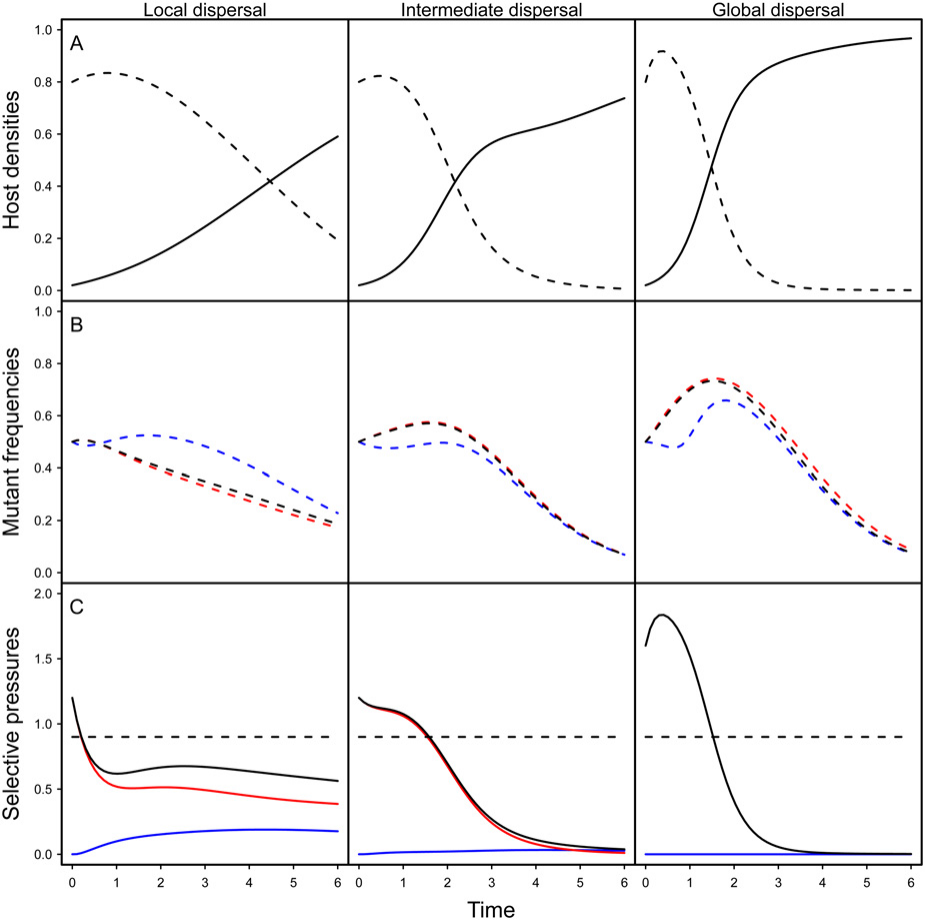
Effect of spatial structure on epidemiology and evolution in the model. The top figures (A) show simulation results for the density of susceptible (dashed line) and infected hosts (full line) for different level of mixing (left: no mixing (*g*_*H*_ = *g*_*P*_ = 0), middle: intermediate (*g*_*H*_ = *g*_*P*_ = 0.5), right: full mixing (*g*_*H*_ = *g*_*P*_ = 1)). The figures in the middle (B) show simulation results for the change in mutant frequency in the total pathogen population (black), in the horizontally infected hosts (red) or in vertically infected hosts (blue) for different levels of mixing. The figures at the bottom (C) indicate the values of the different components of the selection coefficient identified in Eq (1): the horizontal transmission term (red), the vertical transmission component (blue) and the sum of the previous two terms (black) and the magnitude of the cost of virulence (dashed black). Note that the global mutant frequency increases only when the sum of the transmission terms (full black line) is higher than the cost of virulence (dashed line). Our simulations are the result of the numerical integration of the deterministic approximation of the full spatial model, using Improved Pair Approximation (IPA; [31]) with parameters *ϕ* = 1/6 and *θ* = 2/5, which correspond to a triangular lattice (each site has 6 neighbours, see [31] for details). Parameters: *α*_*w*_ = 0.1, *α*_*m*_ = 1, *β*_*w*_ = 2, *β*_*m*_ = 3.5, *δ* = 0.99, *d* = 0.01. Initial conditions: *S*(0) = 0.8, $I_{w}^{H}\left(0\right)=I_{m}^{H}\left(0\right)=0.01$,$I_{w}^{V}\left(0\right)=I_{m}^{V}\left(0\right)=10^{-5}$.

We study a spatial version of the compartmental model depicted in Fig 1. This allows us to make predictions on the epidemiological and evolutionary consequences of spatial structure. First, Fig 2A shows that lower levels of mixing slow down the spread of the infection because in a spatially structured environment, pathogens experience a lower density of susceptible hosts. Second, the change in frequency of a virulent mutant strain is (see Supporting Information Theory in S1 Text):

$$\frac{df}{dt}=f\left(1-f\right)\;\left[\underset{Horizontal\;transmission}{\underset{︸}{\beta_{m}\left[S|I_{m}\right]-\beta_{w}\left[S|I_{w}\right]}}+\underset{Vertical\;transmission}{\underset{︸}{\delta \;b_{I}\left(\;\left[o|I_{m}\right]-\left[o|I_{w}\right]\;\right)}}-\underset{Virulence}{\underset{︸}{\left(\;\alpha_{m}-\alpha_{w}\;\right)}}\right]$$ (1)

The first term measures the contribution of the horizontal transmission route and depends on the local density of susceptible hosts in the neighbourhood of a host infected by a mutant or wild-type parasite The second term measures the contribution of vertical transmission and depends on the local density of empty sites experienced by hosts infected by each type of parasite. Indeed, a vertical transmission event is conditional upon a reproduction event and thus on the availability of empty sites in the neighbourhood. Finally, the last term measures the cost of virulence.

Fig 2B shows numerical simulations based on the pair approximation [29–31] under different levels of mixing (see supporting information Theory in S1 Text). Note that, under all level of mixing, the frequency of the virulent mutant initially increases. Indeed, early on during the epidemics, parasites have access to a large density of susceptible hosts, which favours the spread of more transmissible mutants. This benefit, however, is only transient. As the pool of susceptible hosts is exhausted, more prudent pathogens end up being selected under all mixing treatments. Spatial structure, however, has a dramatic impact on the virulent mutant: less mixing drastically reduces the transient benefit of virulence. To understand this effect, we represent on Fig 2C the effect of spatial structure on the different components of the selection coefficient in Eq (1). Spatial structure acts through two different effects: (i) space reduces the horizontal transmission benefit (red curve in Fig 2C) and (ii) space generates benefit for the virulence mutant through vertical transmission (blue curve in Fig 2C). The first effect is the classical effect of space often discussed as a self-shading or kin selection argument [14,21,32]. The second effect is due to the accumulation of empty sites near virulence mutants, which is caused by the death of hosts infected by related (and thus virulent) genotypes. In other words, virulence mutant have more opportunities to transmit vertically than avirulent genotypes.

**Fig 3:**
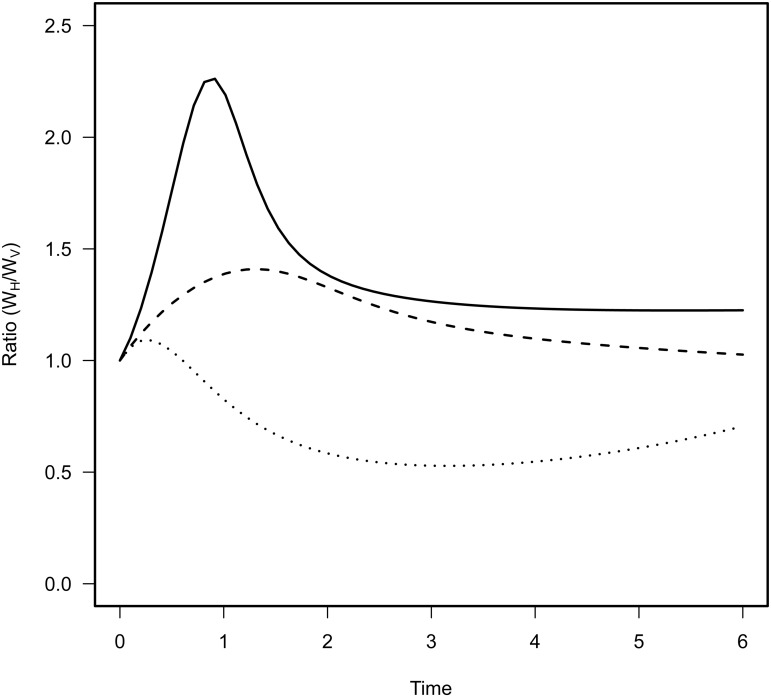
Effect of mixing on the relative contribution of horizontal (W_H_) and vertical transmission (W_V_) to the fitness of the virulent mutant in the model. Each curve corresponds to different levels of mixing: no mixing *g*_*H*_ = *g*_*P*_ = 0 (dotted line), intermediate mixing *g*_*H*_ = *g*_*P*_ = 0.5 (dashed line) and full mixing with *g*_*H*_ = *g*_*P*_ = 1 (full line). The contribution of horizontal transmission is maximal in the early stage of the epidemic because many susceptible hosts are still available. More mixing increases the contribution of horizontal transmission to the fitness of the mutant throughout the epidemics.

In addition, to better grasp the effect of space on pathogen evolution, we track the change in mutant frequency over two types of infected hosts: hosts that acquired the infection horizontally and hosts that acquired the infection vertically. Fig 2B reveals the effect of mixing on the change in frequency of the mutant in these two compartments. In a well-mixed environment the frequency of the mutant is always higher in the horizontally infected hosts. In contrast, in a structured environment, the frequency of the virulent mutant can be higher in vertically infected hosts. This is due to the effect of spatial structure on the opportunities for vertical transmission. As explained above the virulent mutant has more opportunities for vertical transmission because the local density of empty sites is higher (see Fig 2B). Another way to see this effect is to track the ratio W_H_ / W_V_ which provides a measure of the relative contribution of the two routes of transmission to mutant fitness. When this ratio is above 1 the change in mutant frequency is mainly governed by the horizontal transmission route. In contrast, when it is below one the vertical transmission route contributes more to the mutant fitness. Fig 3 shows that during the early stage of the epidemics horizontal transmission drives the evolution of the virulent mutant under all mixing treatments but that the contribution of vertical transmission is always increased by spatial structure.

### Experiment

**Fig 4:**
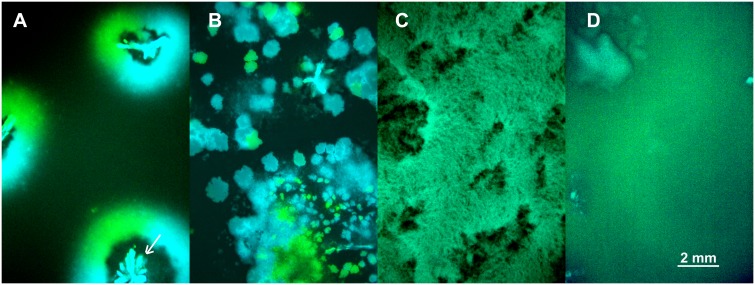
Competition of fluorescently marked virus (λCFP and λYFP—blue and green areas) during invasion into uninfected cells (black areas) at different degrees of mixing (*undisturbed*, *30s*, *24h*, *24h-wet*). **(A)** *Undisturbed environment*—Circular epidemic front segregates into single-virus sectors (blue and green sectors, white arrow depicts the centre of epidemic inoculation). **(B)** *30s disturbance*—Epidemic clusters that are dominated by a single type of virus appear. **(C,D)** Longer periods of disturbance (*24h*) and a liquid surface layer (*24h-wet*) progressively homogenize the spatial genetic structure of virus types.

The above model is an attempt to capture the interplay between the effects of spatial structure and vertical transmission on the evolution of pathogen virulence. To test these predictions experimentally we followed competition of the avirulent λ and virulent λcI857 in spatial epidemics after an initial inoculation of a 1:1 ratio of cells infected by λ or λcI857 onto a biofilm of susceptible cells. Thereupon spatial structure was either left ***undisturbed*** or disturbed for ***30s*** or ***24h*** in the absence of liquid, or 24h in the presence of liquid (***24h-wet***). By engineering fluorescent protein expression cassettes (CFP and YFP) into the viral genome we tagged infected cells by a fluorescent colour. This enabled us to directly visualize pathogen-host structure and spatial prevalence in these four environments (see Fig 4). In the ***undisturbed*** treatment the epidemic spreads in a circular front segregating by viral types (λCFP or λYFP) but large areas remain uninfected (see Fig 4A). In the ***30s*** disturbance treatment the epidemic spreads through the entire biofilm and creates epidemic clusters dominated by a single type of virus (Fig 4B). After ***24h*** disturbance epidemic clusters disappear, but the biofilm remains structured (Fig 4C). In the ***24h-wet*** treatment epidemic structure is completely homogenized (Fig 4D). Thus, as expected from our model simulation (Fig 2A), higher mixing is associated with a significant increase in overall prevalence of infection (χ_1_^2^ = 36.52, p<0.0001, Fig S3 in S1 Text).

**Fig 5:**
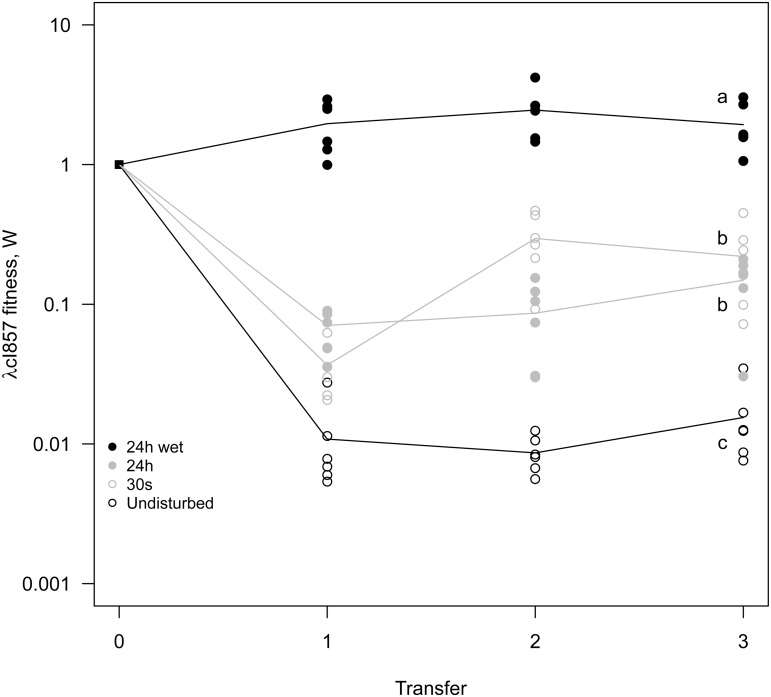
Mixing selects for virulence. A 1:1 mixture of temperate λ and its virulent mutant λcI857 was used to seed spatial epidemics. Spatial structure was disturbed to different degree (***undisturbed*, *30s*, *24h*, *24h-wet***) and transferred onto a fresh biofilm of susceptible hosts for 3 consecutive days. Disturbance of the epidemic structure has a strong effect on the competitive fitness of the virulent λcI857. The ***undisturbed*** environment strongly selects against virulence whereas in a well-mixed environment (***24h-wet***) virulence is beneficial. As expected, intermediate levels of mixing (***30s*, *24h***) lead to intermediate fitness of λcI857. Symbols represent 6 replications for each mixing treatment (with 3 independent replicates for each marker-mutant combination).

The mixing treatment strongly affects competitive fitness *W* of λcI857 (χ_1_^2^ = 79.15, p<0.0001, Fig 5). In the ***undisturbed*** environment λcI857 fitness decreases by ~100 fold after the first transfer day, in contrast in the ***24h-wet*** environment λcI857 fitness increases by ~5 fold. As expected, intermediate spatial disturbance treatments (***30s*** and ***24h***) yield intermediate levels of λcI857 fitness (Fig 5). This demonstrates that erosion of the spatial structure increases the fitness of the virulent λcI857 by ~500 fold. Note that additional transfers did not affect this pattern except for the treatment 30s where λcI857 fitness increases with time (*t* = 5.08, p<10–4).

**Fig 6:**
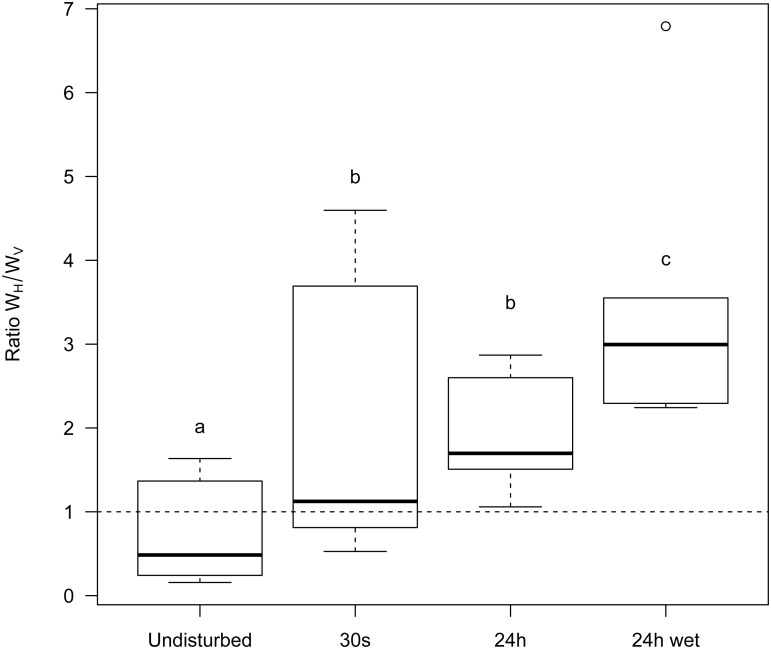
Effect of mixing on the relative contribution of horizontal (W_H_) and vertical transmission (W_V_) to the fitness of the virulent λcI857. More mixing (from ***undisturbed*, *30s*, *24h*** to ***24h-wet***) increases the fitness contribution of horizontal transmission to the competitive ability of λcI857 (W_H_) relative to the fitness contribution of vertical transmission (W_V_) (see Fig 3). Each box represents the median, the first and the third quartiles. Dashed lines delimit 1.5 times the inter-quartile range, above which individual counts are considered outliers and marked as empty circles.

To disentangle the contribution of horizontal and vertical transmission to the fitness of λcI857 we repeated the experiment for the first round of spatial transfer with uninfected cells marked by the red fluorescent protein mCherry. This way we identified new infections from horizontal transmission as doubly coloured cells (mCherry and colour of the new infecting virus) and vertical transmission from the initial lysogen as singly coloured cells (colour of the originally integrated virus). From these data we calculated the competitive fitness of λcI857 among all new infections (horizontal fitness W_H_) and fitness among all vertical transmission events (vertical fitness W_V_) in each spatial environment. Fig 6 presents the effect of mixing on the ratio W_H_ / W_V_ between horizontal and vertical fitness components of λcI857. In the absence of mixing both routes of transmission contribute to λcI857 fitness. Interestingly, in agreement with our theoretical prediction (Fig 2C), we found that higher levels of mixing promote horizontal transmission (χ_1_^2^ = 14,82, p = 0.002). For instance, in the highly mixed environment (***24h-wet*** treatment), 3 out of 4 new infections result from the horizontal transmission route (Fig 6).

## Discussion

During an epidemic, disease prevalence increases, and the opportunities to find uninfected hosts dwindle. This may select for prudent exploitation of the remaining host population. In a previous study, we demonstrated the impact of such epidemiological feedback on the evolution of bacteriophage λ [9]. Here, we use the same experimental system to study the effect of spatial structure on the evolution of bacteriophage λ. To account for latency and vertical transmission in the life cycle of bacteriophage λ, we extend previous theory on the effect of spatial structure on pathogen evolution by allowing for vertical transmission of the pathogen. Our model tries to capture all the details of the bacteriophage λ life-cycle, but for tractability we do not explicitly model the free living stage of the virus. Our central result is that the fitness of a virulent strain relative to a more prudent strain decreases as the environment becomes increasingly structured. A general insight of previous theory is that the outcome of selection on parasite life-history traits depends on the balance between the genetic and epidemiological structures of the parasite population [21]. At the genetic level, parasites infecting a cluster of hosts tend to be related when transmission occurs locally. Hence, competition for the local host supply tends to take place among related pathogens (kin competition) [18,21,32]. At the epidemiological level, local pathogen spread reduces the local density of uninfected hosts, thereby degrading the parasite's local environment and diminishing future opportunities for horizontal transmission [5,14]. From models without vertical transmission, it has been shown that more prudent strategies of host exploitation are selected for if the genetic structure of the parasite population is above a threshold determined by the epidemiological structure of the host population [33]. As interactions become more localized, genetic structure increases and the balance is tilted at the advantage of more prudent strains [21]. We show that vertical transmission does not alter the general theoretical expectation that spatial structure should select for more prudent host exploitation strategies. Yet our analysis reveals that in a spatially structured environment vertical transmission can affect evolutionary dynamics via two main effects. First, vertical transmission affects opportunities of horizontal transmission through a modification of the local density of susceptible hosts (the first term in Eq (1)). Second, vertical transmission generates a force (the second term in Eq (1)) that may counteract the effect of spatial structure. During the early stage of the epidemic this effect remains very low but further work is required to investigate the impact of the effects of vertical transmission on evolutionary stable virulence strategies. Our model is useful to decouple the change in mutant frequency in horizontally and vertically infected hosts. This distinction between different host types is artificial but it helps to grasp the interplay between spatial structure and transmission routes. As in other situations where the pathogen can appear in different states [9] or in different hosts [8, 34–36], tracking the change in frequency between different compartments enables us to generate a prediction regarding the effect of spatial structure on the relative contribution of the two transmission routes on pathogen fitness (Fig 2C).

This new theoretical model generates predictions that we tested experimentally with bacteriophage λ. Unlike our previous experimental study in chemostats [9], the present experiment has been realized on agar plates to manipulate the amount of spatial structuring. Yet, on a normal agar plate bacterial growth eventually enters stationary phase and the epidemiological and evolutionary dynamics halt. Consequently, our experiments allow us to test predictions during the initial phase of the epidemics only. During this phase we made three qualitative predictions that we verified experimentally. First, we confirmed that lower levels of mixing reduce the speed of the epidemic. Second, we confirmed that a virulent strain is outcompeted by a more prudent strain when the environment becomes increasingly structured. This is our main result and it agrees well with previous experimental studies realized with horizontally transmitted pathogens [22–23]. Third, our experimental setup allows monitoring the relative contribution of horizontal and vertical transmission among new infections and confirms that mixing decreases the contribution of vertical transmission to pathogen fitness.

The evolution of host exploitation strategies can often be explained at the level of within-host selection. For parasites competing for within-host resources, more prudent host exploitation should be selected for when the probability of multiple infections decreases [4]. In our model, superinfection exclusion by a latent λ prophage guarantees that only related pathogen individuals compete within the same host cell and, hence, limits selection for increased replication and virulence at the within-host level. Similarly, many covertly infecting and genome-integrating viral pathogens take within- host competition out of the equation by active exclusion of superinfection. In herpes and retroviruses, for example, superinfection exclusion is well described and might be a key requirement for the maintenance of viral latency [37–39]. Yet some mutations are known to enable λ to break through superinfection exclusion [26]. We did not observe such mutants in our present experiments, but we were able to extend our theory to explore the effect of spatial structure on the epidemiology and evolution of such virulence mutants that can superinfect already infected bacteria (see supporting information Theory in S1 Text). This analysis shows that mixing does affect the epidemiology and evolution in this alternative scenario: more mixing enhances the spread of the virulence mutant. Hence, this analysis suggests that the effect of spatial structure on the transient dynamics we report in the present study is robust to this modification of the life cycle. We believe that similar predictions may be feasible to understand and predict the epidemiology and evolution of a broad range of pathogens with complex transmission modes.

## Methods

### Theory

The theoretical analysis is presented in the supporting information (S1 Text). We examine the evolutionary dynamics of a vertically and horizontally transmitted pathogen in a spatially structured environment. The analytical model reveals that the change in frequency can be decomposed in different terms that characterize the local environment of competing variants of the pathogens. These terms can be tracked in numerical simulations (Fig 2). Further understanding of the evolutionary dynamics can be obtained by focusing on the change in frequency of a mutant pathogen in hosts that have acquired the pathogen horizontally (*I*_*H*_) and in hosts that have acquired the pathogen vertically (*I*_*V*_).

### Experiments

Host exploitation strategies of λ and λcI857 were quantified by three independent assays. **(1) Virus production (PFU/mL)**: Lysogens were grown to OD_600nm_ = 0.6 at 30°C and shifted to 35°C for 2h until lysis occurred. Viral titers were determined by qPCR (Roche LightCycler480 primers F:5’AATGAAGGCAGGAAGTA3’ R:5’GCTTTCCATTCCATCGG3’) on filtered lysates (Millipore 96-well filtration plate Multiscreen HTS). CP values were calibrated to PFU/ml by top-agar plating a dilution series of a λvir (3x10^9^ pfu). **(2) Growth of infected hosts (CFU/mL)**: Lysogens were diluted to OD_600nm_ = 0.07 and grown for 6h at 35°C in eight replicates in 96-well plates (900 rpm, Titramax shaker (Heidolph, Germany)). Final OD_600nm_ was measured in an Infinity200 microplate reader (Tecan, Austria). OD_600nm_ values were calibrated to CFU/ml by plating a dilution curve. **(3) Lysogenization rate** was determined by challenging non-infected E.coli MG1655 of OD_600nm_ = 0.1 with 10^8^ PFU/mL free virus particles of λCFP, λYFP, λcI857CFP and λcI857YFP for 24h. After 24h, the proportion of lysogenized (fluorescent) cells was determined by flow cytometry (Fig S2 in S1 Text).

Fluorescently labelled phage were constructed as described in [9]. Spatial epidemics were started by spreading a 10^8^ cfu/ml suspension of uninfected E.coli MG1655 (starved 24h in 0.2%Maltose, 10mM MgSO_4_) onto agar plates until dried. Thereafter a 1:1 mixture of cells infected by λ and λcI857 was inoculated in localized spots by an array of 0.4mm stainless steel tattooing needles at about 4mm distance. Epidemic structure was disturbed by rolling 4mm sterile stainless steel beads over the dry agar surface for *30s* or *24h* or over agar surface humidified by sterile saline solution (*24h-wet*) or kept *undisturbed*. Agar plates were incubated at 35°C in agitation (250rpm), but only in the *24h* and *24h-wet* treatment steel beads remained on the agar surface. To control for marker effects all competitions were carried out with reciprocal marker-mutant combinations (λYFP vs. λcI857CFP and λCFP vs. λcI857YFP). Spatial structure was photographed on an Olympus BH-2 RCF microscope with a 2x fluorite objective and filter cube #69380 (Chroma Thechnology Corp., VT, USA). For the daily transfer of the epidemic structure the needle array described above was poked into plates that had been incubated for 24 hours and subsequently the needle array was poked into agar plates containing a layer of uninfected cells as described above. For flowcytometry the bacterial layer was washed off with 2 ml saline and analysed on a B&D Fortessa flowcytometer (Excitation CFP, YFP, mCherry at 405, 488 and 561 nm).

**Horizontal and vertical transmission** was estimated by repeating the spatial epidemics for a single cycle with lysogens of λCFP, λYFP, λcI857CFP and λcI857YFP carrying pBAD18 and uninfected E.coli MG1655 carrying plasmid pRSET-mCherry. 20μg/ml Ampicillin was added to the medium to prevent spontaneous plasmid loss. Horizontal transmission events were scored by double coloration (mCherry+CFP and mCherry+YFP). Horizontal and vertical fitness were calculated as mentioned above by only considering mCherry+ or mCherry- cells.

**The relative fitness of λcI857** was calculated as *W* = (*f*_t_ /(1- *f*_t_))/ (*f*_0_ /(1- *f*_0_)) where *f*_t_ measures the frequency at time *t*. In general the frequency was calculated over the whole host population. In Fig 6 we distinguish horizontal fitness, W_H_, and vertical fitness, W_V_, by focusing either on the frequency in newly infected bacteria (bacteria carrying the plasmid pRSET-mCherry) or on the frequency in previously infected bacteria (bacteria that do not carry the plasmid pRSET-mCherry), respectively. In Fig 4 we present the ratio W_H_ / W_V_ for different mixing treatments.

### Statistical analysis

Analyses were carried out using the R statistical package (version 3.0.2). The fitness W of λcI857 (Fig 5, S1 Data) was log transformed prior to fitting in a mixed effects model (lme, nlme package). Mixing (undisturbed, 30 s, 24h and 24h-wet) and transfer day (1, 2 and 3) were fitted as fixed effects while population was nested within the marker effect (the two reciprocal marker-mutant combinations) to account for the repeated measurements at different points in time. The ratio W_H_ / W_V_ (Fig 6, S1 Data) was fitted in a mixed effect model with mixing as a fixed factor and marker as a random effect. The prevalence of the infection (Fig S3 in S1 Text and S1 Data) was fitted in a mixed effect model with mixing as a fixed factor and marker colour as a random effect. Maximal models, including all higher-order interactions, were simplified by sequentially eliminating non-significant terms and interactions to establish a minimal model. The significance of explanatory variables in mixed effect models was established using a likelihood ratio test which is approximately distributed as a chi-square distribution [40]. A posteriori contrasts were carried out at the final time point of the experiments in Figs 5, 6 and S3 (in S1 Text) by aggregating factor levels together and by testing the fit of the simplified model using a likelihood ratio test [41].

## Supporting Information

S1 Text Theoretical appendix, extended simulation results and supporting experimental results. (PDF)

S1 Data Experimental data for Figs 5, 6 and S3 (in S1 Text). (TXT)
