# Supplementary material for: Spatial Structure, Transmission Modes and the Evolution of Viral Exploitation Strategies
Source: PLoS Pathog. 2015 Apr 21;11(4):e1004810. doi: 10.1371/journal.ppat.1004810 (PMC4405370; doi:10.1371/journal.ppat.1004810)
Supplement: S1 Text — (PDF) [file ppat.1004810.s001.pdf]

Supplementary Online Material to "Spatial structure, transmission modes and the evolution of viral exploitation strategies"

T. Berngruber, S. Lion & S. Gandon

January 31, 2015

1 Theory

1.1 Epidemiological dynamics with vertical transmission

We consider a regular lattice of sites. Each site can be either empty (o) or occupied by one individual host. Hosts can be either susceptible (S) or infected (I). Susceptible hosts die at rate d, while infected hosts die at rate $d+\alpha$. Reproduction or transmission events can occur either locally or globally. Susceptible hosts reproduce at rate b_S and infected hosts reproduce at rate b_I. With probability g_H, a host can reproduce in a random empty site, while with probability $1-g_H$, reproduction takes place in a random empty site in the neighbourhood of the reproducing individual. With probability δ, the offspring is born infected (vertical transmission).

Similarly, an infected host can transmit the disease horizontally at rate β, either globally, with probability g_P, to a random susceptible host in the population, or locally to a random susceptible neighour, with probability $1-g_P$.

The dynamics of the average global densities of susceptible (p_S) and infected (p_I) hosts can thus be described by the following (exact) system of equations

$$\frac{dp_S}{dt} = b_S[o|S]p_S + (1-\delta)b_I[o|I]p_I - dp_S - \beta[S|I]p_I \quad (1)$$

$$\frac{dp_I}{dt} = \beta[S|I]p_I + \delta b_I[o|I]p_I - (d+\alpha)p_I \quad (2)$$

where

$$[o|x] = (1-g_H)q_{o/x} + g_H p_o$$

is the effective density of empty sites experienced by a random host in state x. This effective density is equal to p_o, the global density of empty sites, if $g_H = 1$, and is equal to $q_{o/x}$, the local density of empty sites in the neighbourhood of a x host, if $g_H = 0$. Similarly, we use the notation

$$[S|I] = (1-g_P)q_{S/I} + g_P p_S$$

for the effective density of susceptible hosts experienced by an infected host.

1.2 Evolutionary dynamics with vertical transmission

1.2.1 Evolution of virulence

To model evolutionary dynamics we allow two parasite strains to compete. First, we consider a latent (wild-type) strain w, and a virulent (mutant) strain m. As in our experiment we consider a situation where the mutation affects the survival (and the transmission rate) of infected hosts but not their

fecundity. This yields

$$\frac{dp_S}{dt} = b_S[o|S]p_S + (1-\delta)b_I([o|I_w]p_{I_w} + [o|I_m]p_{I_m}) - dp_S - \beta_w[S|I_w]p_{I_w} - \beta_m[S|I_m]p_{I_m} \tag{3}$$

$$\frac{dp_{I_w}}{dt} = \beta_w[S|I_w]p_{I_w} + \delta b_I[o|I_w]p_{I_w} - (d+\alpha_w)p_{I_w} \tag{4}$$

$$\frac{dp_{I_m}}{dt} = \beta_m[S|I_m]p_{I_m} + \delta b_I[o|I_m]p_{I_m} - (d+\alpha_m)p_{I_m} \tag{5}$$

The change in frequency $f = p_{I_m}/(p_{I_w} + p_{I_m})$ of mutant parasites can be written as

$$\frac{df}{dt} = f(1-f)(\lambda_w - \lambda_m) \tag{6}$$

where λ_i is the per-capita growth rate of strain i. Hence

$$\frac{df}{dt} = f(1-f)\left[(\beta_m[S|I_m] - \beta_w[S|I_w]) + \delta b_I([o|I_m] - [o|I_w]) - (\alpha_m - \alpha_w)\right] \tag{7}$$

Equation (7) allows us to identify three forces affecting the change in frequency of the mutant. First, the term $-(\alpha_m - \alpha_w)$ represents the cost of virulence: the frequency of the mutant strains tends to decrease if the mutant parasite causes higher host mortality upon infection. Second, horizontal transmission favours the mutant strain if the net number of secondary infections produced through this route by an individual infected host is greater than that of the wild-type parasite. Third, vertical transmission may also increase the frequency of mutant parasites if hosts infected by the mutant parasite have access to more empty sites than those infected by the wild-type.

When infection and reproduction are global ($g_H = g_P = 1$), equation (7) collapses to

$$\frac{df}{dt} = f(1-f)\left[(\beta_m - \beta_w)p_S - (\alpha_m - \alpha_w)\right] \tag{8}$$

Hence, the reproduction of infected hosts (and therefore vertical transmission) has no direct contribution to the change in frequency of the mutant. However, the density of susceptible hosts depends on the fecundity of infected hosts, b_I, and on the fidelity of vertical transmission, δ. In particular, at endemic equilibrium, vertical transmission is expected to increase prevalence and reduce the density of susceptible hosts. Through this effect, vertical transmission affects the balance between the benefit (horizontal transmission) and the cost (reduced survival) of virulence and selects for lower virulence. This is the clasical effect of the transmission mode on virulence evolution (Frank, 1996). Away from the endemic equilibrium, the effect of vertical transmission can be more complex (Day & Gandon, 2007; Lélu et al., 2013). Equation (7) shows that spatial structure adds yet another level of complexity. When infection and reproduction are local ($g_H = g_P = 0$), equation (7) can be written as

$$\frac{df}{dt} = f(1-f)\left[(\beta_m q_{S/I_m} - \beta_w q_{S/I_w}) + \delta b_I(q_{o/I_m} - q_{o/I_w}) - (\alpha_m - \alpha_w)\right] \tag{9}$$

Hence, spatial structure leads to an additional selective force of vertical transmission. Now the evolutionary outcome depends on the availability of empty sites around hosts infected by the mutant or wild-type strain because more empty sites mean more opportunities for vertical transmission.

1.2.2 Decoupling vertical and horizontal transmissions

Instead of tracking the change in densities of the total pool of hosts infected by each strain, we now split the infected class into two compartments, those infected hosts that are produced through horizontal transmission (I_w^H and I_m^H), and those that are produced through vertical transmission (I_w^V and I_m^V).

The resulting dynamics are as follows

$$\frac{dp_{I_w^H}}{dt} = \beta_w[S|I_w]p_{I_w} - (d+\alpha_w)p_{I_w^H} \tag{10}$$
$$\frac{dp_{I_w^V}}{dt} = \delta b_I[o|I_w]p_{I_w} - (d+\alpha_w)p_{I_w^V} \tag{11}$$
$$\frac{dp_{I_m^H}}{dt} = \beta_m[S|I_m]p_{I_m} - (d+\alpha_m)p_{I_w^H} \tag{12}$$
$$\frac{dp_{I_m^V}}{dt} = \delta b_I[o|I_m]p_{I_m} - (d+\alpha_m)p_{I_m^V} \tag{13}$$

where I_w and I_m represent the pooled classes of infected hosts infected by the wild-type or mutant strain, respectively (e.g. $p_{I_w} = p_{I_w^H} + p_{I_w^V}$). From these equations, we easily derive the change in the mutant frequency among infected hosts for each transmission routes:

$$\frac{df_H}{dt} = f_H(1-f_H)\left[(\beta_m[S|I_m] - \beta_w[S|I_w]) - (\alpha_m - \alpha_w)\right] \tag{14}$$
$$\frac{df_V}{dt} = f_V(1-f_V)\left[\delta b_I([o|I_m] - [o|I_w]) - (\alpha_m - \alpha_w)\right] \tag{15}$$

Thus, because the cost of virulence is the same for each class, the distribution of the mutant in each class will mainly depend on the respective magnitude of the net transmission vs. net fecundity. Note that, if the fidelity of vertical transmission is zero (infected hosts produce susceptible hosts), the frequency of mutant parasites in vertically infected hosts drops to zero, as expected. In a well-mixed population, this is always the case, even when the fidelity of vertical transmission is high.

1.2.3 Evolution with superinfection

We also consider another mutation that affects the ability to superinfect an already infected bacteria. In bacteriophage λ several mutations have been described that allow to resist superinfection inhibition. These mutations usually act by weakening the affinity of the *oLoR* operator for the *cI* repressor of the resident phage (Ptashne, 1992; Berngruber et al., 2010). A side effect of these mutations is to lose the ability to induce lysogeny. In other words, the ability to superinfect bacteria (and thus to have additional opportunities for horizontal transmission) are traded-off against the ability to transmit vertically. The change in frequency of the mutant type then becomes

$$\frac{df}{dt} = f(1-f)\Big[\sigma_m\beta_m([I_w|I_m] + [I_m|I_w]) + (\beta_m[S|I_m] - \beta_w[S|I_w]) \\ + \delta b_I((1-\varepsilon_m)[o|I_m] - [o|I_w]) - (\alpha_m - \alpha_w)\Big]$$

where σ_m measures the ability to superinfect an already infected bacteria (relative to an uninfected bacteria) and θ_m measures the fecundity reduction of bacteria infected by the superinfection mutant (relative to a bacteria infected by the wild-type). Note that both σ_m and θ_m are expected to be between 0 and 1. We recover equation (7) when $\beta_m = \varepsilon_m = 0$.

A full analysis of the evolutionary dynamics under this scenario is beyond the scope of the present study because the change in frequency of the mutations we are following are not superinfection mutants. Yet, we explored an extreme situation where $\beta_m = \varepsilon_m = 1$. This case corresponds to a mutant that can superinfect all infected cells but gives up completely vertical transmission. Examples if such mutants have been identified in bacteriophage λ (Ptashne, 1992). Figure S1 shows the results of numerical simulations similar to the ones presented in Figure 2 in the main text. First, we see that mixing speeds up the epidemic and reduces the total bacteria density (Figure S1A). The reduction of total host density is due to the castration of the bacteria infected by the virulent mutant. Second, we recover the effect of mixing on the spread of the virulent mutant (Figure S1B). Very low level of mixing can prevent the growth of a mutant that is fully able to superinfect already infected bacteria. As soon as there is some level of global transmission, however, this mutant outcompetes the wild-type. Third, our analysis allows us to decompose the fitness of the mutant into four different components

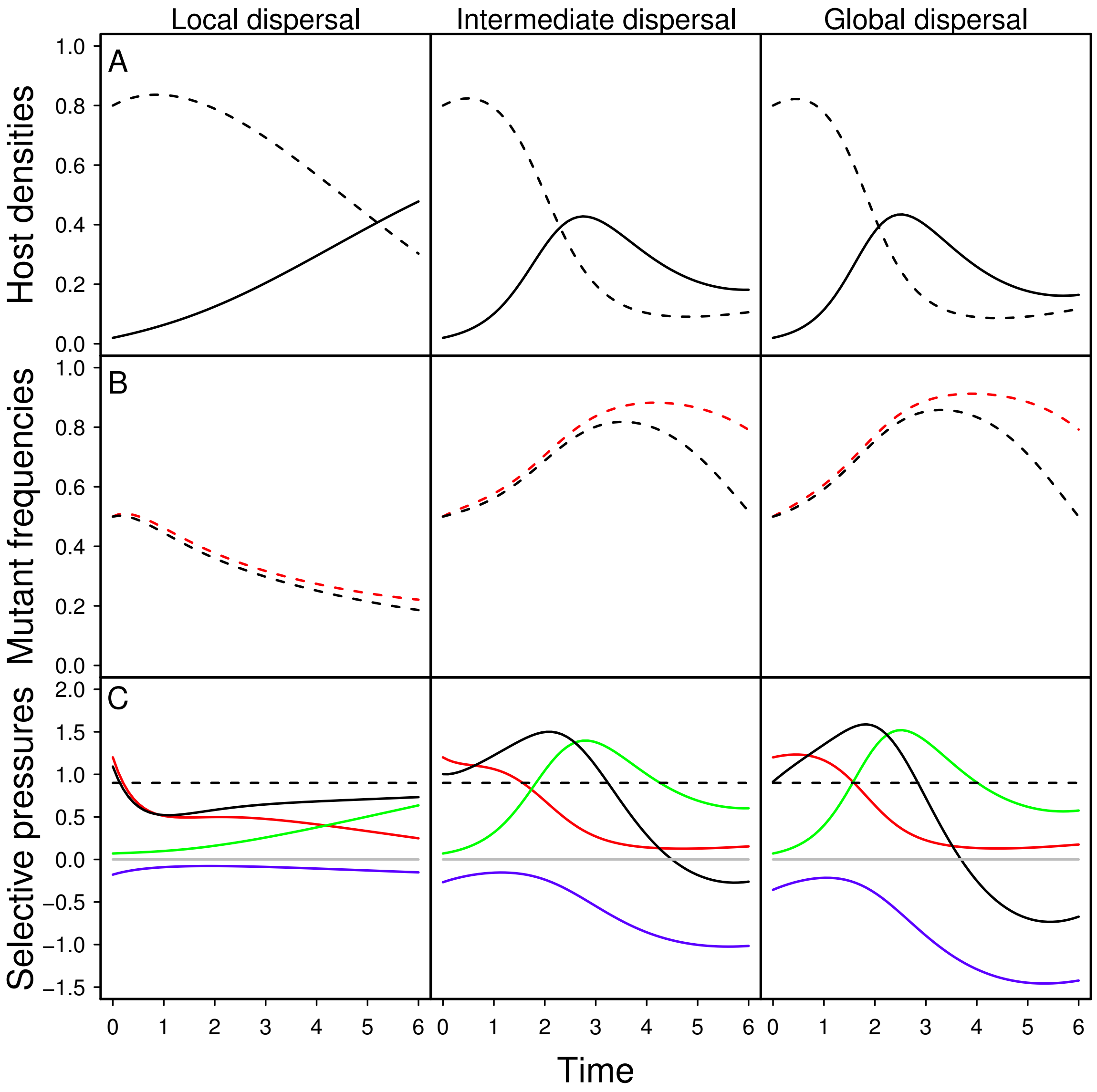

Figure S1: **Effect of spatial structure on epidemiology and evolution in the model with superinfection.** The top figures show simulation results for the density of susceptible (dashed line) and infected hosts (full line) for different level of mixing (on the left: no mixing, in the middle: intermediate, on the right: full mixing). The figures in the middle show simulation results for the change in mutant frequency in the total pathogen population (black) in the horizontally infected hosts (red) or in vertically infected hosts (blue) for different levels of mixing. The figures at the bottom indicate the values of the different components of the selection coefficient identified in equation (16): the horizontal transmission term (red), the vertical transmission component (blue), the superinfection component (green), the sum of the previous three terms (black) and the magnitude of the cost of virulence (dashed black). Note that the global mutant frequency increases only when the transmission terms (full black line) are higher than the cost of virulence (dashed line). Parameters: $\alpha_w = 0.1$, $\alpha_m = 1$, $\beta_w = 2$, $\beta_m = 3.5$, $\delta = 0.99$, $d = 0.01$. Our numerical simulations are the result of the numerical integration of the deterministic approximation of the full spatial model, using Improved Pair Approximation (IPA; van Baalen, 2000) with parameters $\phi = 1/6$ and $\theta = 2/5$, which correspond to a triangular lattice (each site has 6 neighbours, see van Baalen, 2000 for details).

(Figure S1C). We recover the three components identified in and ploted in Figure 2C in the main text. Note that the fitness component related to reproduction becomes negative in this scenario because the mutant is castrating its host and is not verticaly transmited. Yet, a fourth fitness component emerges through superinfection (green curve). This term drives the evolution of the system when most bacteria are infected towards the end of the epidemic and most importantly when there is some mixing. As in the previous scenario it is difficult to discuss the long term evolution of this system. Further studies are needed to evaluate the evolutionary stability of virulence strategies with vertical transmission and superinfection. Yet, this analysis demonstrates that our theoretical approach can be extended to a broad range of scenarios and shows that the effect of spatial structure on the evolution of virulence is robust when superinfection is considered.

2 Experiments (figures S2 and S3)

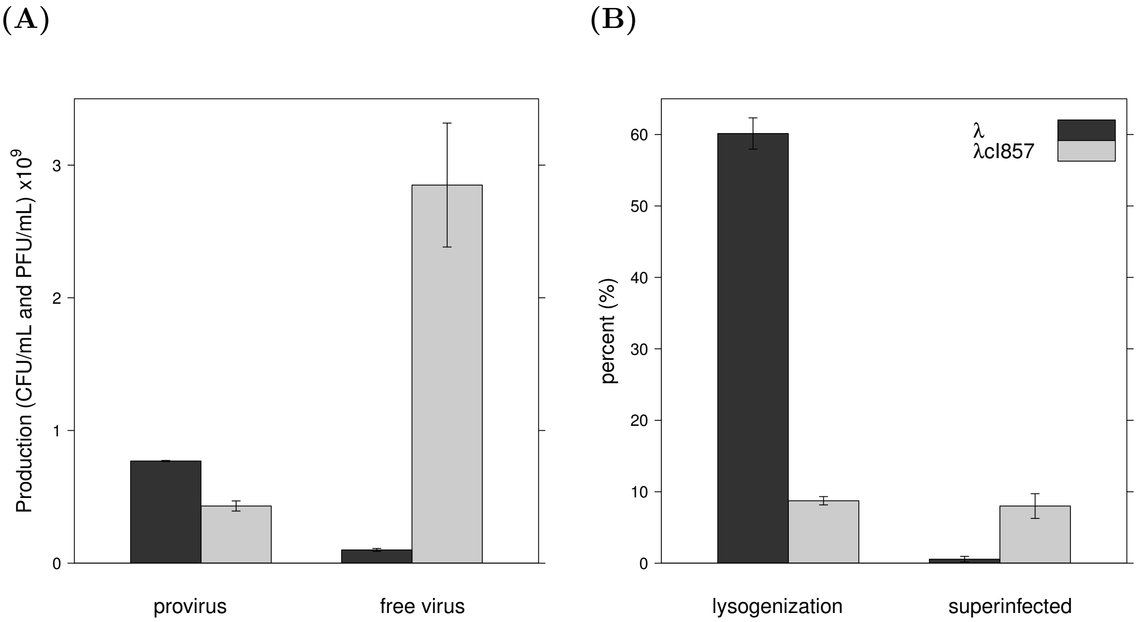

Figure S2: Host exploitation strategies of λ and λcI857. (a) Production of infected cells carrying a provirus copy (CFU/ml) and production of free virus particles (PFU/ml) by λcI857 and λ within 6 h of growth of a lysogen culture at 35∘C. Note that λI857 produces 10 times more free virus particles at the expense of the survival of infected (provirus carrying) host cells. (b) Lysogenization rate of λcI857 and λ and rate at which a lysogen of one strain is superinfected by free virus particles of the opposite strain (Data in (a) redrawn from the data obtained in Berngruber et al. (2013)).

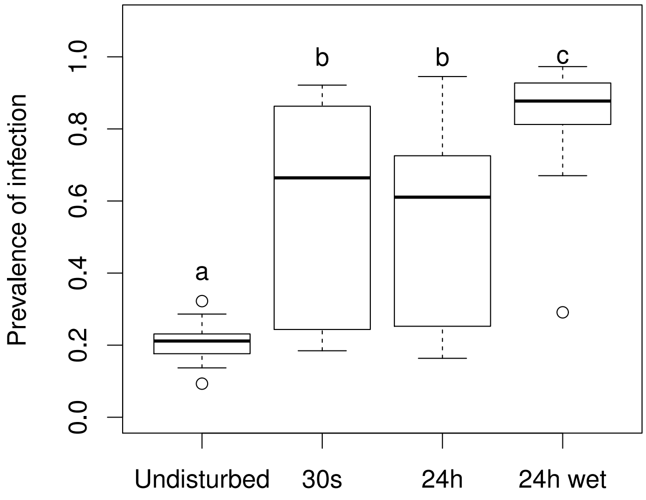

Figure S3: Total prevalence increases with disturbance of the environment (undisturbed, 30 s, 24 h and 24 h-wet). We plot the prevalence measured as the fraction of fluorescent bacteria (i.e. bacteria carrying a wildtype or a mutant prophage) at the end of day 3 of the competition experiment presented in Figure 5 in the main text.

References

Ptashne, M. (1992). *A Genetic Switch: Phage Lambda and Higher Organisms.* Blackwell Publishers, Oxford, UK.

Frank, S. A. (1996). Models of parasite virulence. *Q. Rev. Biol.* **71**(1): 37–78.

Van Baalen, M. (2000). "Pair approximations for different spatial geometries." In: *The Geometry of Ecological Interactions: Simplifying Spatial Complexity.* Ed. by Dieckmann, U., Law, R. & Metz, J. A. J. Cambridge University Press, Cambridge, pp. 359–387.

Day, T. & S. Gandon (2007). Applying population-genetic models in theoretical evolutionary epidemiology. *Ecol. Lett.* **10**: 876–888. DOI: 10.1111/j.1461-0248.2007.01091.x.

Berngruber, T. W., F. J. Weissing & S. Gandon (2010). Inhibition of superinfection and the evolution of viral latency. *Journal of Virology.* **84**(19): 10200–10208. DOI: 10.1128/JVI.00865-10.

Berngruber, T. W., S. Lion & S. Gandon (2013). Evolution of suicide as a defense strategy against pathogens in a spatially structured environment. *Ecol. Lett.* **16**: 446–453. DOI: 10.1111/ele.12064.

Lélu, M., M. Langlais, M.-L. Poulle, E. Gilot-Fromont & S. Gandon (2013). When should a trophically and vertically transmitted parasite manipulate its intermediate host? The case of Toxoplasma gondii. *Proc. R. Soc. B.* **280**: 20131143. DOI: 10.1098/rspb.2013.1143.
